# MicroRNA Involvement in Allergic and Non-Allergic Mast Cell Activation

**DOI:** 10.3390/ijms20092145

**Published:** 2019-04-30

**Authors:** Irit Shefler, Pazit Salamon, Yoseph A. Mekori

**Affiliations:** 1 The Herbert Mast Cell Disorders Center, Laboratory of Allergy and Clinical Immunology, Meir Medical Center, Kfar Saba 4428164, Israel; irit.shefler@clalit.org.il (I.S.); pazit.salamon@clalit.org.il (P.S.); 2 Sackler School of Medicine, Tel Aviv University, Tel Aviv 6997801, Israel; 3 Tel Hai College, Tel Hai 1220800, Israel

**Keywords:** microRNA, mast cells, inflammation

## Abstract

Allergic inflammation is accompanied by the coordinated expression of numerous genes and proteins that initiate, sustain, and propagate immune responses and tissue remodeling. MicroRNAs (miRNAs) are a large class of small regulatory molecules that are able to control the translation of target mRNAs and consequently regulate various biological processes at the posttranscriptional level. MiRNA profiles have been identified in multiple allergic inflammatory diseases and in the tumor microenvironment. Mast cells have been found to co-localize within the above conditions. More specifically, in addition to being essential in initiating the allergic response, mast cells play a key role in both innate and adaptive immunity as well as in modulating tumor growth. This review summarizes the possible role of various miRNAs in the above-mentioned processes wherein mast cells have been found to be involved. Understanding the role of miRNAs in mast cell activation and function may serve as an important tool in developing diagnostic as well as therapeutic approaches in mast cell-dependent pathological conditions.

## 1. Introduction

**Figure 1:**
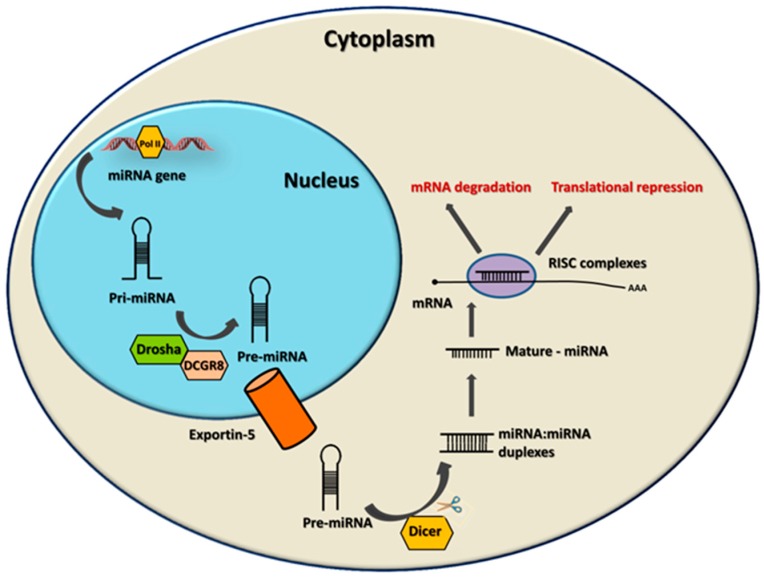
Biogenesis of microRNA (miRNA). miRNAs genes are transcribed as primary miRNAs (pri-miRNAs) by RNA polymerase II (Pol II) in the nucleus. The pri-miRNAs are cleaved by the Drosha–DGCR8 complex to produce a precursor miRNA (pre-miRMA), which is then exported to the cytoplasm by the exportin-5-RNA GTP complex. In the cytoplasm, the pre-miRMA is further processed by Dicer to a short RNA duplex. One strand is degraded, while the mature miRNA is incorporated into the RNA-induced silencing complex (RISC), which leads to targeting an mRNA, leading to its degradation or the repression of translation.

MicroRNAs (miRNAs) are a class of short single-stranded RNA molecules that are only 19 to 25 nucleotides long that serve as negative regulators of protein expression. They bind to the 3’untranslated region (3′UTR) of their target mRNA through at least six to eight-nucleotide long complementary sequences. Expression of the miRNAs is often tissue-specific and developmentally controlled. They mediate posttranscriptional gene silencing of the target gene mainly through the degradation of target mRNAs or the inhibition of translation [1,2]. Thus, they are able to regulate a wide variety of functions, such as proliferation, differentiation, apoptosis, stress response, and immune response [3,4]. MiRNAs usually belong to families, which consist of evolutionarily related members that partially share sequences and targets [5]. In the human genome, there are approximately 239 different miRNAs families, which together express more than 2000 different mature miRNAs. A particular miRNA can have hundreds of target genes, and an individual gene is usually targeted by multiple miRNAs. Thereby, miRNAs collectively have been suggested to influence the expression of approximately 30% of genes [1]. MiRNAs are transcribed in the nucleus by RNA polymerase II and III [6]. Nuclear processing by the enzyme Drosha produces a pre-miRNA transcript that can be shuttled into the cytoplasm. Final production of the mature miRNA species require further cytoplasmic processing by the RNAase III enzyme Dicer producing a 19–25 base pair product that is capable of being incorporated into the RNA-induced silencing complex (RISC) that contains the component Argonaute (Ago2) protein (Figure 1) [7]. MiRNAs are widely recognized as modulators of many parts of the immune system. Unique miRNA expression profiles are found in the cells of the adaptive and innate immune systems, regulating lineage commitment, proliferation, effector functions, and differentiation in normal and diseased conditions [8,9].

Mast cells (MC) are derived from hematopoietic progenitor cells that enter nearly all vascularized tissues, where they complete their maturation. They are considered to play a key role in the immune system, both in innate and adaptive immunity. MC are regarded as key effector cells in immediate hypersensitivity reactions and allergic disorders such as cutaneous/mucosal allergy, asthma, atopic dermatitis, hypersensitivity, anaphylaxis, and mastocytosis [10,11,12,13].

MC are well-recognized by two kinds of highly expressed cell surface receptors: FcεRI, which is the IgE receptor, and c-kit (CD117), which is the stem cell factor (SCF) receptor. Upon cell activation, a variety of bioactive mediators that are pre-stored in cytoplasmic granules, including histamine, heparin, proteases, proteoglycan, and antimicrobial peptides, are rapidly released into the extracellular milieu. MC can also selectively produce and release newly synthesized potent inflammatory mediators such as leukotriene C4 (LTC4), prostaglandin D2 (PGD2), and cytokines/chemokines [13,14,15]. The interaction of IgE with its receptor results in the phosphorylation of spleen tyrosine kinase (Syk), calcium (Ca^2+^) influx, and the activation of protein kinase C (PKC), mitogen-activated protein kinase (MAPK), and nuclear factor (NF-κB) [16].

MC can also be activated via several FcεRI-independent mechanisms including direct injury, microbial products that signal through Toll-like receptors (TLRs), some polybasic compounds known as basic secretagogues, peptides, dextran, complement, IgG-antigen complexes, proteases, cytokines, and chemokines, endogenous mediators within the nervous system such as neurotensin and somatostatin, as well as neuropeptides such as substance P [17,18,19,20]. MC can also be activated by interaction with activated T cells or their microvesicles [21]. We have previously shown that microvesicles derived from activated T cells (mvT*) can stimulate MC to degranulate and release several cytokines that are specific to this activation pathway, such as IL-24 and Oncostatin M (OSM) [22].

Despite the important roles that both MC and miRNAs play in the immune response, little is known on the biological effects of miRNA on MC function. This review will focus on recent findings that are relevant to the effect of miRNA on MC function in both allergic and non-allergic immune responses.

## 2. Expression of miRNAs in MC

Silencing of Dicer activity, which is a key enzyme of the miRNA biogenesis, leads to total repression of endogenous miRNAs [23]. To elucidate whether degranulation in human MC is regulated by miRNAs, MC degranulation was measured after silencing of the Dicer expression. Indeed, Dicer repression, by transfection of siRNA, led to the total repression of endogenous miRNAs and reduced FcεRI-mediated MC degranulation. Thus, this suggests that miRNAs are involved in affecting MC activation [24].

Several expression profiling studies revealed miRNAs’ expression patterns in MC. The first study by Monticelli et al. assessed 181 miRNAs in hematopoietic cell types, including bone marrow-derived MC (BMMCs). They have demonstrated that BMMCs expressed high levels of miR-26a, miR-24, and miR-27a, and low levels of miR-223 compared to other hematopoietic lineages [25]. In a more recent study, several other miRNAs that were upregulated or downregulated in DNP-stimulated BMMCs compared to resting MC have been demonstrated. Cluster analysis revealed that a total of 17 miRNAs were significantly differentially expressed in the activated MC compared to resting cells. Among these altered miRNAs, seven were upregulated and 10 were downregulated. Notably, miR-21a and miR-3113 were the most remarkably upregulated and downregulated miRNAs in activated MC, respectively. Analysis of the potential targets of part of those miRNAs revealed a significant alternation of mRNA expression levels of some key signaling molecule genes such as Lyn, Scamp1, Vav3, and Csf2 [26]. More recently, the miRNAs expression profile of DNP-stimulated mouse BMMCs compared to untreated cells was also detected. Thirteen miRNAs were found to be upregulated, and seven miRNAs were downregulated in DNP-stimulated BMMCs compared to resting BMMCs. A selected miRNA among the upregulated miRNAs, let7i, was shown to inhibit MC degranulation by suppressing Exoc8 expression, which is an exocytosis-related gene [27].

## 3. The Involvement of miRNAs in MC Activation Pathways

### 3.1. Involvement of miRNAs in IgE-Mediated Allergic Diseases

During allergic inflammation, MC activation occurs by FcεRI cross-linking with specific antigens. This leads to an immediate phase of IgE-associated hypersensitivity reaction by releasing histamine and other mediators. During the late-phase reaction, MC favors the recruitment of eosinophils and neutrophils through the production of different cytokines and chemokines. Upon prolonged or repetitive exposure to specific antigens, the activation of MC becomes sustained, leading to chronic allergic inflammation.

Several miRNAs have been found to regulate different genes that participate in this MC activation pathway, thereby influencing MC function in different allergic disorders.

The overexpression of miR-142-3p was found to enhance FcεRI-mediated degranulation in human MC and in murine BMMCs. LPP (lipoma preferred (translocation) partner) was found to be a potential target of this miRNA. LPP regulates actin filament assembly by binding to α-actinin and to vasodilator-stimulated phosphoprotein (VASP) involved in actin polymerization. Actin cytoskeleton regulates the association between cross-linked IgE–FcεRI and raft components. The inhibition of actin polymerization increases degranulation in MC. Therefore, it was suggested that LPP is associated with MC degranulation by organizing the actin filament [24].

MC activation is associated with activation and phosphorylation of a member of the MAPKs extracellular signal-regulated kinase (ERK) [28]. MiR-126 positively regulates MC proliferation and FcεRI-mediated cytokine production such as IL-6, tumor necrosis factor-α (TNF-α), and IL-13 via enhancing ERK activity by inhibiting Sprouty-related, EVH1 domain containing 1 (Spred1) expression, which is known as a negative regulator of ERK [29]. A previous study had also revealed the involvement of miR-126 in airway hyperresponsiveness induced by house dust mites [30]. Exploring the effect of this miRNA on MC activation through IgE demonstrated that miR-126 accelerated IgE-mediated MC degranulation, which was associated with the phosphoinositide 3-kinase (PI3K)/protein kinase B (Akt) signaling pathway and enhanced Ca^2+^ influx [31]. In accordance with this observation, several studies have also demonstrated the participation of the PI3K/Akt signaling pathway in the regulation of MC degranulation by miRNAs. For example, miR-155 controlled MC activation by FcεRI cross-linking via modulating the PI3Kγ signaling pathway, which results in increased degranulation and cytokine release [32]. MiR-223 has also been identified as an important modulator of degranulation and cytokine release in MC. The downregulation of miR-223 promoted MC degranulation [33] and IL-6 release [34] via the PI3K/Akt pathway by targeting insulin-like growth factor 1 receptor (IGF1R), which is a known activator of many downstream kinases, including Akt [35].

Another miRNA that was upregulated in response to the activation of MC by IgE is miR-132. The analysis of predicted targets of this miRNA revealed that miR-132 regulated the growth factor heparin-binding EGF-like growth factor (HB–EGF) expression. As HB-EGF is also upregulated in MC activation; the authors suggested that miR-132 serves as a negative regulator of this MC-derived growth factor that may take part in silencing the activated MC, and thus contributes to avoiding the prolonged stimulation of the tissue environment in chronic allergen exposure [36].

MiR-221-222 was significantly upregulated in MC stimulation, and regulated proliferation and cell cycle checkpoints in MC in response to acute stimulation [37]. Furthermore, this study shed light on the miR-221-222 transcriptional regulation mechanism that enables miR-221-222 to regulate the cell cycle through partially inhibiting p27^Kip1^ protein expression after MC stimulation. Additionally, in response to MC stimulation through FcεRI cross-linking, miR-221 had several MC-specific, activation-dependent functions that affected the extent of degranulation, cytokine production such as that of IL-6 and TNF-α, and cell adherence [38]. MiR-221 was also found to be upregulated in a murine asthma model and in murine MC after stimulation with lipopolysaccharide (LPS). Increased miR-221 expression led to IL-4 secretion from these cells by regulating the activation of phosphatase and tensin homolog (PTEN), which induced the activation of p38 and NFκB [39].

Another miRNA that was found to regulate NFκB activation in MC is miR-302e, which is a dominant member of the miR-302 family. MiR-302e was found to be an important regulator of allergic inflammation in human MC. The expression of miR-302e was significantly decreased in response to MC activation by phorbol 12-myristate 13-acetate (PMA) and calcium ionophore (A23187) or ovalbumin (OVA). The overexpression of this miRNA blocked PMA/A23187-induced or OVA-induced increase in inflammatory cytokine levels, such as IL-1β, IL-6, TNF-α, and thymic stromal lymphopoietin (TSLP), while miR-302e inhibition further promoted the release of these cytokines. MiR-302e is a novel miRNA that negatively regulates RelA expression, which is also known as p65, and is a member of the NFκB family, suggesting that miR-302e ameliorates allergic inflammation at least in part through inhibition of the NFκB signaling pathway [40].

MiR-20a is an additional miRNA that was downregulated in response to the activation of MC by PMA/A23187. The overexpression of miR-20a in activated MC resulted in the inhibited production of pro-inflammatory cytokines such as TNF-α, IL-1β, and IFN-γ, and in contrast promoted IL-10 production. Histone deacetylase 4 (HDAC4) was found to be the potential target of this miRNA. MiR-20a targeted HDAC4 and suppressed its expression, which contributed to the epigenetic regulation of IL-10 expression [41].

In murine allergic airway inflammation models, the upregulation of miR-21 correlated with reduced expression of its predicted target IL-12p35, which is a salient cytokine for T-lymphocyte polarization, resulting in decreased IL-12 production and increased Th2 responses, eosinophilia, and allergic airway inflammation, suggesting that miR-21 may regulate a number of processes involved in allergic airway inflammation [42]. Mir-21 was also found to mediate the inhibition of MC degranulation by inhibiting the p38 pathway in allergic contact dermatitis in rats [43].

MiR-143 directly targets IL-13Ra1 and suppresses the expression of IL-13Ra1 in human MC, resulting in a downregulation of allergic responses [44].

An additional miRNA that was found to be involved in the regulation of inflammation and other processes in innate as well as adaptive immune responses is miR-146. MiR-146a is one member of a family comprising miR-146a and miR-146b, and is often more abundantly expressed in the immune system. Its expression is increased with the maturation and activation of the immune cells [45,46]. MiR-146a targeted TNF receptor-associated factor 6 (TRAF6) and IL-1-receptor associated kinase (IRAK1), which are two adaptor molecules that are implicated in the NFκB activation pathway [47,48]. Thus, miR-146a was shown to act as a negative regulator of NFκB activation, more specifically, working in a feedback system in which a stimulus induces NFκB activation through a MyD88-dependent pathway, resulting in the expression of inflammatory genes, but also in the upregulation of miR-146a. In turn, this downregulates IRAK1 and TRAF6 expression, thereby reducing the activity of this pathway [48]. In MC and in other immune cells such as monocytes and T cells, miR-146a expression in response to a variety of stimuli appeared to be exquisitely dependent on NFκB p50. A lack of this NFκB family member strongly impaired the activation-induced expression of miR-146a in response to the activation of MC by the IgE–antigen complex or LPS [46]. Indeed, in the absence of NFκB p50, MC showed altered tissue homeostasis and survival due to the increased expression of pro-survival factors such as Bcl-2 and A1, as well as the reduced expression of pro-apoptotic factors such as Bax, but also of miR-146a, which in this context acted by reducing MC survival [46].

### 3.2. Involvement of miRNAs in Non-Allergic Diseases

MC can be activated via several FcεRI- independent mechanisms. One of the activation pathways by which MC have been found to be activated is by interaction with activated T cells or by their microvesicles [21,22]. The observations of close physical proximity between MC and T lymphocytes in inflamed tissues have raised the possibility of a functional interaction between these two cell populations. Indeed, morphologic studies have documented an increase in the local density of MC and their activation during T cell-mediated inflammatory processes, as observed in cutaneous delayed-type hypersensitivity [49,50]. The physical contact of MC with activated T cells, or with T cell-derived microvesicles (mvT*), is associated with Ras activation and sustained ERK phosphorylation, leading to MC degranulation and mediator release [28,51,52,53]. These microvesicles may act as intercellular carriers and may deliver bioactive proteins and RNA to recipient cells, including mRNAs [54] and miRNAs [55,56], which can affect their properties. One of the miRNA that was found to be delivered to MC by microvesicles released from activated T cells is miR-4443. Indeed, the stimulation of MC with mvT* led to the overexpression of miR-4443. MiR-4443 serves as a negative regulator of the Protein Tyrosine Phosphatase Receptor type J (PTPRJ) gene, which is known to modulate the Ras signaling pathway by the dephosphorylation of ERK [57]. Indeed, the overexpression of miR-4443 was found to augment ERK phosphorylation and IL-8 release in MC activated by mvT*. Thus, by delivering miR-4443, T cell-derived microvesicles may play an important role in MC activation within T cell-mediated inflammatory processes where MC were found to be involved.

MiR-155 is known to be a major contributor to inflammatory diseases, such as atopic dermatitis, allergic airway inflammation, passive cutaneous anaphylaxis, and rheumatoid arthritis [58,59,60]. MiR-155 was found to regulate allergic airway inflammation by modulating Th2 responses through the transcription factor PU.1 [61]. Moreover, the selective blockade of the PI3K pathway inhibited degranulation in miR-155-/- BMMCs, suggesting that miR-155 controlled MC activation by FcεRI via the PI3K pathway [32].

Many stimuli elicit MC responses; among them is the cytokine IL-33, which can be produced by several cells such as endothelial cells, epithelial cells, fibroblasts, keratinocytes, and MC themselves, in response to damage or stress, and promotes a Th2 response [62,63,64]. The binding of IL-33 to the ST2/IL-1RacP receptor on MC results in the release of several cytokines, chemokines, and lipid mediators [62,63]. The activation of MC by IL-33 was decreased following treatment with lactic acid, which is a known participant in inflammatory environments [65]. This inhibition involved the selective suppression of miR-155. Moreover, the overexpression of this miRNA was found to abolish the suppressive effects of lactic acid [66]. The expression of miR-155 in IL-33-treated peritoneal MC isolated from allergic contact dermatitis rats was increased in comparison to IL-33-treated naive MC, resulting in the increased production of inflammatory cytokines such as IL-6 and TNF-α. These results indicate a positive correlation between miR-155 expression and cytokine production in allergic contact dermatitis [67].

IL-10 can enhance MC activation and exacerbate anaphylaxis. The treatment of MC with IL-10 has been shown to augment FcεRI-mediated cytokine production in a Stat3-dependent manner. Stat3 exerts its activity through the induction of miR-155, which suppresses SOCS1, a suppressor of cytokine signaling 1, resulting in enhanced cytokines production [60].

### 3.3. MiRNAs Involvement in MC–Tumor Interaction

In addition to their role in allergic inflammation, MC also serve as a component of the tumor microenvironment, and are often found at the site of tumors. They have been attributed both to pro-tumorigenic and anti-tumorigenic roles depending on the tumor type and its developmental stage [68]. MC promotes cancer growth by the secretion of angiogenic factors, tissue remodeling, and modulation of the host immune response. The anti-tumorigenic actions of MC include direct growth inhibition, immunologic stimulation, and decreased cell mobility [68,69]. The interaction between tumor cells and MC may be achieved by releasing tumor-derived extracellular vesicles (exosomes, microvesicles) into the extracellular space, which interact with the recipient cells to transfer their content. A variety of biomolecules such as proteins, DNA, and RNA including mRNAs [54] and miRNAs [55,56] are present in these extracellular vesicles, and can affect the properties of the recipient cells [70,71]. Xiong et al. [72] have demonstrated that MC activated by hepatitis C virus E2 envelope glycoprotein (HCV-E2) released exosomes that contain high levels of miR-490. The transfer of this miRNA through exosomes to hepatocellular carcinoma cells resulted in the inhibition of the EGFR/AKT/ERK pathway, which in turn led to the inhibition of tumor metastasis.

The expression profiling of miRNAs related to MC activation and angiogenesis in salivary gland tumors revealed a significant negative correlation of miR-9 expression with microvessel density [73]. MiR-9 has also been found to be highly transcribed in high-grade canine cutaneous MC tumors (MCT) and to contribute to the invasive phenotype and spontaneous metastasis of malignant MC [74].

There is a bidirectional relationship between tumor cells and MC. For example, condition medium derived from tumor cells increased the expression of several transcription factors in MC that influence allergic inflammation, such as Histone deacetylase-3 (HDAC3), SOCS1, and SNAIL, as well as increasing MC degranulation. MiR-122 was found to prevent the influence of the tumor-derived condition medium on MC activation by targeting SOCS1. MiR-122 also prevented the condition medium of activated MC from enhancing the invasion and migration potential of B16F1 tumor cells, thus indicating the above-mentioned bidirectional effect [75].

## 4. Concluding Remarks

MiRNAs are critical regulators of immune responses and immunological disorders by modulating cell differentiation, proliferation, survival, and the effector function, as well as the resolution of an immune response. The advent of miRNAs has potentially revealed a new level of complexity to be considered for every biological process. Research in the past decade uncovered many of the details regarding miRNA biogenesis and regulation, but the functions of miRNAs in relation to allergic inflammation are still largely unexplored.

**Figure 2:**
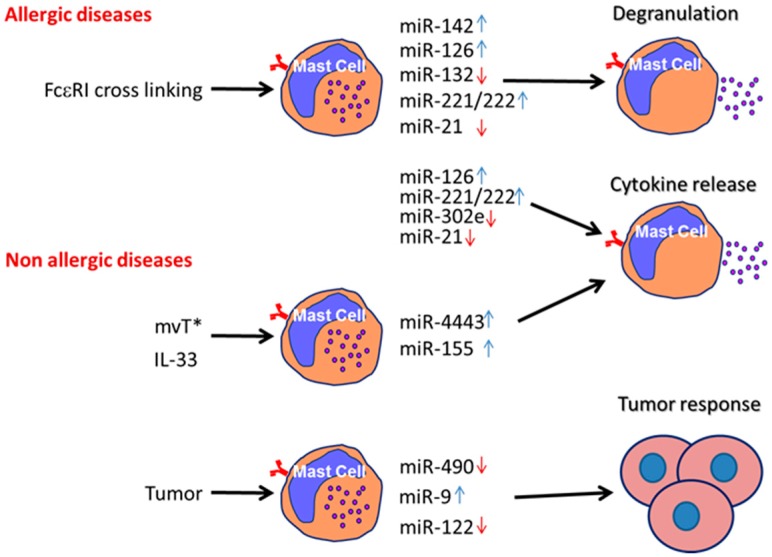
MiRNA’s effect on mast cell activation. Red arrows indicate decreased mast cell activation by miRNAs. Blue arrows: increased activation of the mast cell.

This review summarizes the possible roles of various miRNAs in different pathways of MC activation and function, including IgE-mediated allergic response, non-allergic response such as activation by mvT* or by several cytokines, and at the site of malignant tumors (Figure 2). MiRNAs act both as inducers or suppressors of MC activation and function depending on MC triggers, origin, and inflammatory disorders by affecting different targets participating in MC activation (see more details in Table 1). Thus, certain miRNAs may be considered as a potential therapeutic approach in the management of inflammatory processes where MC have been found to participate. Clearly, miRNAs are potential noninvasive biomarkers, and the modulation of their expression can be used for therapeutic purposes.

## Figures and Tables

**Table 1:** miRNAs’ function in mast cell activation and proliferation.

MiRNA	Trigger	miRNA Effect on Mast Cells	Target	Ref
miR-142	FcεR I *	Increases degranulation	LPP	[24]
miR-126	FcεRI *	Regulates proliferation, degranulation, and cytokine production	Spred1, PI3K	[29,31]
miR-223	FcεRI *	Decreases degranulation and IL-6 release	IGF1R	[33,34]
miR-132	FcεRI *	Decreases activation	HB-EGF	[36]
miR-221/222	FcεRI *	Regulates proliferation and cell cycle Increases degranulation and cytokine production; reduces migration.	p27^Kip1^PTEN	[37,38,39]
miR-302e	FcεRI *, PMA/Ion *	Decreases cytokine secretion	RelA	[40]
miR-20a	PMA/Ion **	Inhibits production of pro-inflammatory cytokines	HDAC4	[41]
miR-21	allergic inflammation	Inhibits degranulation and IL-12 production	IL-12p35 P38	[42,43]
miR-143	allergic inflammation	Downregulates allergic responses	IL-13Ra1	[44]
miR-146	FcεRI *	Reduces activation	TRAF6, IRAK1	[46,47,48]
miR-4443	mvT * #	Increases ERK phosphorylation and IL-8 release	PTPRJ	[57]
miR-155	FcεRI * IL-33 IL-10	Increases degranulation and cytokines Increases production of cytokines Increases production of cytokines	PI3K -- SOCS1	[32,60,66,67]
miR-490	HCV-E2	Inhibits tumor metastasis	EGFR/AKT/ERK pathway	[72]
miR-9		Increases invasion of neoplastic MC		[74]
miR-122	Tumor response	Decreases activation	SOCS1	[75]

* FcεRI cross-linking; ** A23187-Ca^2+^ Iononophore. # mvT*-T cell derived microvesicles.

## Abbreviations

A23187	Calcium ionophore
Ago2	Argonaute
BMMCs	Bone marrow derived mast cells
ERK	Extracellular signal-regulated kinase
HCV-E2	Hepatitis C virus E2 envelope glycoprotein
HDAC3	Histone deacetylase-3
IGF1R	Insulin-like growth factor 1 receptor
IRAK1	IL-1-receptor associated kinase
LPP	Lipoma preferred (translocation) partner
LTC4	leukotriene C4
MAPK	Mitogen-activated protein kinase
MC	Mast cells
MCT	Canine cutaneous MC tumors
MicroRNAs	miRNAs
mvT*	T cell-derived microvesicles
NF-κB	Nuclear factor-κB
OSM	Oncostatin M
PGD2	Prostaglandin D2
PI3K	Phosphoinositide 3-kinase
PMA	Phorbol 12-myristate 13-acetate
PTEN	Phosphatase and tensin homolog
PTPRJ	Protein Tyrosine Phosphatase Receptor type J
RISC	RNA-induced silencing complex
SCF	Stem cell factor
SOCS1	Suppressor of cytokine signaling 1
Spred1	Sprouty-related, EVH1 domain containing 1
SYK	Spleen tyrosine kinase
TLRs	Toll like receptors
TRAF6	TNF receptor-associated factor 6
TSLP	Thymic stromal lymphopoietin
VASP	Vasodilator-stimulated phosphoprotein
